# MRI-Based Nomogram of Prostate Maximum Sectional Area and Its Zone Area for Prediction of Prostate Cancer

**DOI:** 10.3389/fonc.2021.708730

**Published:** 2021-09-09

**Authors:** Shaoqin Jiang, Zhangcheng Huang, Bingqiao Liu, Zhenlin Chen, Yue Xu, Wenzhong Zheng, Yaoan Wen, Mengqiang Li

**Affiliations:** ^1^Department of Urology, Changhai Hospital, Second Military University, Shanghai, China; ^2^Laboratory of Urology, Department of Urology, Fujian Union Hospital, Fujian Medical University, Fuzhou, China

**Keywords:** nomogram, prostate maximum sectional area, prostate zone area, prostate cancer, prostate biopsy

## Abstract

**Objective:**

To reduce unnecessary prostate biopsies, we designed a magnetic resonance imaging (MRI)-based nomogram prediction model of prostate maximum sectional area (PA) and investigated its zone area for diagnosing prostate cancer (PCa).

**Methods:**

MRI was administered to 691 consecutive patients before prostate biopsies from January 2012 to January 2020. PA, central gland sectional area (CGA), and peripheral zone sectional area (PZA) were measured on axial T2-weighted prostate MRI. Multivariate logistic regression analysis and area under the receiver operating characteristic (ROC) curve were performed to evaluate and integrate the predictors of PCa. Based on multivariate logistic regression coefficients after excluding combinations of collinear variables, three models and nomograms were generated and intercompared by Delong test, calibration curve, and decision curve analysis (DCA).

**Results:**

The positive rate of PCa was 46.74% (323/691). Multivariate analysis revealed that age, PSA, MRI, transCGA, coroPZA, transPA, and transPAI (transverse PZA-to-CGA ratio) were independent predictors of PCa. Compared with no PCa patients, transCGA (AUC = 0.801) was significantly lower and transPAI (AUC = 0.749) was significantly higher in PCa patients. Both of them have a significantly higher AUC than PSA (AUC = 0.714) and PV (AUC = 0.725). Our best predictive model included the factors age, PSA, MRI, transCGA, and coroPZA with the AUC of 0.918 for predicting PCa status. Based on this predictive model, a novel nomogram for predicting PCa was conducted and internally validated (C-index = 0.913).

**Conclusions:**

We found the potential clinical utility of transCGA and transPAI in predicting PCa. Then, we firstly built the nomogram based on PA and its zone area to evaluate its diagnostic efficacy for PCa, which could reduce unnecessary prostate biopsies.

## Introduction

Prostate cancer (PCa) is the most common cancer among men in the Western world, and it has an increasing prevalence (1). There is an international consensus that early detection and treatment of PCa can improve the survival rate of PCa patients. Prostate-specific antigen (PSA) is the most widely used screening marker to detect PCa at an early stage. The larger clinical trial found that patients having undergone PSA screening had 25% lower PCa death rates than those who did not (2). After tests reveal an elevated serum PSA level, most patients require puncture biopsy of the prostate, because the prostate biopsy remains the gold standard method for diagnosing PCa. However, we have to face a clinical problem that the prostate biopsy is an invasive operation. It not only brings pain and fear to the patients, but also may cause medical complications such as infection and hemorrhage (3). Because prostate biopsy always has the probability of missing tumor tissue, it is not able to make a 100% diagnosis of PCa. The rate of negative prostate biopsies was substantially high (58.51%–69.30%) especially in cases with only elevated PSA levels, thus greatly affecting patients’ quality of life (4, 5). Therefore, it is rational to avoid the biopsy on patients who are ultimately proved to be negative cases.

In order to overcome the limitations of PSA test, Benson et al. proposed the concept of PSA density (PSAD, PSA value divided by prostate volume), which was considered to increase the accuracy of PSA test for diagnosing the PCa (6). The main principle is that PCa tissues can release more PSA per unit volume to blood serum than enlarged or normal prostate tissues do. Recent research had also shown that PSA density could overweigh PSA in distinguishing clinically significant PCa and intraprostatic inflammation before prostate biopsy (7). However, it has been reported that the prostate volume was frequently roughly calculated using the prolate ellipsoid formula before operation, in which there is 10%–20% error compared with prostatectomy specimens in the clinical situation (8, 9). So, this kind of prostate volume should be further improved for assessing the exact risk of PCa. Therefore, we considered finding new MRI-based predictors to enhance the role of roughly calculated prostate volume for predicting PCa.

In MRI images of prostate zonal anatomy, the prostate comprises the peripheral zone, transition zone, central zone, and anterior fibromuscular stroma (10). A related study showed that approximately 75%–85% of PCa cases are located in the peripheral zone, rather than the central gland. The central gland is the typical site of BPH, which includes the transition zone, central zone, and anterior fibromuscular stroma (11). MRI images can clearly identify different anatomical areas of the prostate, which is beneficial to improve the detection rate of PCa. We first propose the concept of the prostate maximum sectional area (PA) for predicting PCa by MRI images, which includes both central gland sectional area (CGA) and peripheral zone sectional area (PZA). Comparatively, MRI is regarded as the most precise noninvasive method, as it can assess PA with high reproducibility and accuracy compared with rough prostate volume calculated by the common formula (12).

Nomogram is a simple intuitive graph of a complex mathematical formula (13). It is widely used for cancer prediction, primarily because of their ability to use biologic and clinical variables building a graphically depictive statistical predictive model that is tailored to an individual patient (14). User-friendly graphical interfaces for generating these estimates facilitate the use of nomograms to aid in clinical decision-making.

The purpose of the current study was to establish a new nomogram about PA and its associated zone area such as CGA and PZA on axial T2 fat-saturated MRI for diagnosing PCa. To the best of our knowledge, no previous literature has employed MRI-based PA and the associated zone area for the prediction of PCa before prostate biopsy among Chinese population.

## Methods

### Study Population

**Figure 1:**
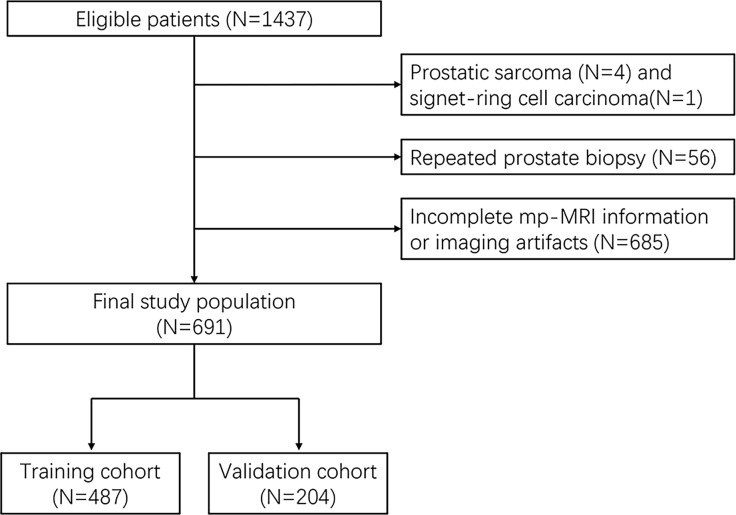
Flow chart of patient selection.

The study was designed as a retrospective cohort study that was conducted in the Laboratory of Urology and the Department of Urology of Fujian Medical University Union Hospital (Fuzhou, China). We enrolled 691 consecutive patients who underwent multiparametric magnetic resonance image (mp-MRI) before initial transrectal ultrasound (TRUS)-guided prostate biopsy from January 2012 to January 2020, followed by data anonymization. Eligible patients who matched the selection criteria were identified by the following criteria: elevated PSA levels (≥10 ng·ml^−1^), suspected cancer on digital rectal examination (DRE), hyperechoic or hypoechoic TRUS, or abnormal MRI findings. For PSA values between 4 and 10 ng·ml^−1^, the biopsy criterion was ratio of free to total PSA < 16%. The exclusion criteria were as follows: previous prostate biopsy, history of prostate surgery, pathological examination revealing tumors other than adenocarcinoma, and incomplete mp-MRI information or imaging artifacts (**Figure 1**). The study was ethically approved by the Institutional Review Board of Fujian Medical University Union Hospital with an approval number of 2020KY059. Written informed consent was obtained from patients before the study commenced. Details of patients’ identity had to be omitted. Our work complies with the Code of Ethics of the World Medical Association (Declaration of Helsinki, revised in 2013).

### Clinical Data and Variable Definitions

Clinical characteristics including age, body mass index (BMI), PSA, free PSA (FPSA), free-to-total PSA (FTPSA), prostate volume (PV), PSA density (PSAD), MRI, transverse prostate maximum sectional area (transPA), coronal prostate maximum sectional area (coroPA), sagittal prostate maximum sectional area (sagiPA), transverse peripheral zone sectional area (transPZA), coronal peripheral zone sectional area (coroPZA), sagittal peripheral zone sectional area (sagiPZA), transverse central gland sectional area (transCGA), coronal central gland sectional area (coroCGA), sagittal central gland sectional area (sagiCGA), alkaline phosphatase (ALP), and lactate dehydrogenase (LDH) were collected before prostate biopsy. Subsequently, transverse PSA-to-PA ratio (transPSAPA), coronal PSA-to-PA ratio (coroPSAPA), sagittal PSA-to-PA ratio (sagiPSAPA), transverse PSA-to-PZA ratio (transPSAPZA), coronal PSA-to-PZA ratio (coroPSAPZA), sagittal PSA-to-PZA ratio (sagiPSAPZA), transverse PSA-to-CGA ratio (transPSACGA), coronal PSA-to-CGA ratio (coroPSACGA), sagittal PSA-to-CGA ratio (sagiPSACGA), transverse PZA-to-CGA ratio (transverse prostate area index, transPAI), coronal PZA-to-CGA ratio (coronal prostate area index, coroPAI), and sagittal PZA-to-CGA ratio (sagittal prostate area index, sagiPAI) were calculated. The prostate maximum sectional area on prostate T2WI MRI had the following definition: In the transverse plane, when the bilateral prostate lobes are basically symmetrical, and the quasi-circular internal urethral sphincter can be seen in the middle of the prostate, the maximum section is the one for which the sectional area becomes smaller when scanning upward or downward. In the coronal plane, when the bilateral prostate lobes are basically symmetrical, and the strip-type internal urethral sphincter can be seen in the middle of the prostate, the maximum section is the one for which the sectional area becomes smaller when scanning upward or downward. In the sagittal plane, when the strip-type internal urethral sphincter can be seen in the middle of the prostate, the maximum section is the one for which the sectional area becomes smaller when scanning upward or downward.

### Image Acquisition and Interpretation

A Siemens Magnetom Trio Tim 3.0-T superconducting MRI scanner with an 18-channel phased-array torso coil was used to create all magnetic resonance images [repetition time (TR) 400 ms, echo time (TE) 80 ms, slice thickness = 3 mm, interslice gap = 30%, acquisition four times with fat-suppression technique]. T2-weighted images in the sagittal, coronal, and transverse planes, diffusion-weighted images, apparent diffusion coefficient in the transverse plane, and dynamic contrast-enhanced images were acquired according to the international prostate MRI guidelines (15). Interpretation of the MRI findings was performed by a radiologist and a urologist (with 5 or more years of experience in prostate imaging),who measured PA and CGA on fat-saturated T2WI MRI (**Figure S1**).

### Prostate Biopsy Method

Following local non-infiltrative anesthesia, all prostate biopsies were performed transrectally under TRUS guidance (BK Medical, USA). A standard 13-core systematic prostate biopsy was obtained including transitional, peripheral, and anterior zone from base to apex by an 18-gauge/25-cm biopsy needle (Bard Peripheral Vascular, Inc). All patients underwent standard prostate biopsies, which were performed by an experienced urologist (more than 5 years of experience in prostate biopsy). All biopsy specimens were examined and recorded by two experienced pathologists.

### Statistical Analysis

**Figure 2:**
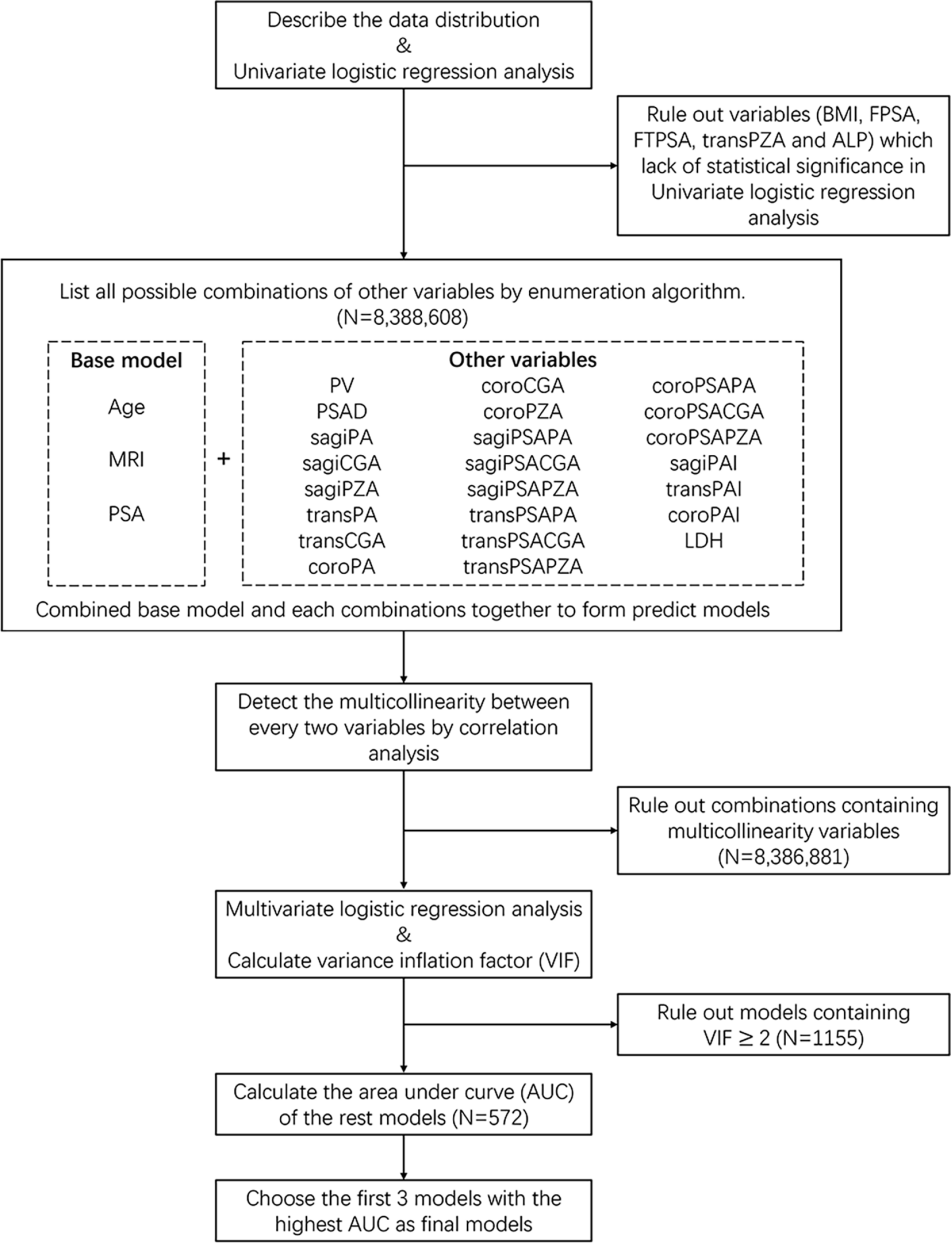
Flow chart of statistical analysis.

Distributions of variables were compared by the chi-squared test for categorical variables and the Mann–Whitney *U* test for continuous variables, which was not normally distributed. The values of all continuous variables (age, BMI, PSA, FPSA, FTPSA, transPA, coroPA, sagiPA, transPZA, coroPZA, sagiPZA, transCGA, coroCGA, sagiCGA, transPSAPA, coroPSAPA, sagiPSAPA, transPSAPZA, coroPSAPZA, sagiPSAPZA, transPSACGA, coroPSACGA, sagiPSACGA, ALP, and LDH) were not normally distributed. Variables including BMI, FPSA, FTPSA, transPZA, and ALP were excluded due to lack of statistical significance in univariate logistic regression analysis. We integrated variables with great clinical significance including age, PSA, and MRI into the base model. The remaining variables were reassembled into all kinds of possible combinations through enumeration algorithm. Then, we combined base model and each different combinations together to form our predict models. Correlation analysis was used to detect the multicollinearity between every two variables (**Figure S2**). Any model that contained two or more multicollinearity variables will be eliminated before the next step. Then, multivariate logistic regression analysis was performed on the rest of these models to identify the independence of each predictor for diagnosing PCa and calculate its variance inflation factor (VIF). Models will also be eliminated when their VIF ≥ 2. The diagnostic efficacy of these models was evaluated by the area under the curve (**Figure 2**). The first three combinations with the highest AUC were chosen as our final models. The statistical differences among three models and each single predictor were compared by Delong test, respectively. The cutoff value, sensitivity, specificity, and positive and negative likelihood ratios were computed for these variables and prediction models. Nomograms were generated to predict the probability of PCa, based on the multivariate regression coefficients in three models. These models were recalibrated both in the training cohort and the validation cohort to evaluate the nomogram’s discrimination capacity by 1,000 random bootstrap samples with replacement. Calibration slope less than 1 reflects proper fit of the model. The clinical utility of three models was quantified by decision curve analysis (DCA) through summing the benefits (true positives) and subtracting the harms (false positives). Statistical significance was defined as *p*-value < 0.05. Statistical analysis, nomogram, and calibration plot were generated using R studio (version 4.0.3).

## Results

### Clinical Characteristics

A total of 230 (47.23%) of 487 patients in the training cohort and 93 (45.59%) of 204 patients in the validation cohort were diagnosed with PCa (**Table 1**). Univariate logistic regression analysis showed that all variables were statistically significant predictors of PCa detection except for BMI, FPSA, FTPSA, transPZA, and ALP in the training cohort. No significance was found in variables between the training cohort and validation cohort except for age (**Table 2**).

**Table 1:** Clinical characteristics of patients before the prostate biopsy.

	Training cohort				Validation cohort			
	No PCa	PCa	OR (95% CI)	*p*-value	No PCa	PCa	OR (95% CI)	*p*-value
	(*n* = 257)	(*n* = 230)			(*n* = 111)	(*n* = 93)		
Age*	67 (62;73)	70 (64;76)	1.05 (1.02;1.07)	<0.001	69 (64;74)	72 (66;77)	1.03 (1.00;1.07)	0.023
BMI*	23.00 (21.50;24.91)	23.88 (21.35;25.99)	1.04 (0.98;1.10)	0.082	23.90 (22.50;25.43)	22.70 (20.52;25.50)	0.92 (0.84;1.01)	0.031
MRI:				<0.001				<0.001
Abnormal	92 (35.8%)	186 (80.9%)	Ref.		43 (38.7%)	76 (81.7%)	Ref.	
Normal	165 (64.2%)	44 (19.1%)	0.13 (0.09;0.20)		68 (61.3%)	17 (18.3%)	0.14 (0.07;0.27)	
FPSA*	1.53 (0.92;2.50)	1.68 (0.69;7.27)	1.11 (1.06;1.17)	0.101	1.49 (0.98;2.42)	1.33 (0.18;3.51)	1.09 (1.02;1.16)	0.411
PSA*	11.13 (7.44;17.91)	26.09 (9.54;97.90)	1.04 (1.03;1.05)	<0.001	11.42 (7.07;16.74)	29.38 (13.22;86.47)	1.05 (1.03;1.07)	<0.001
FTPSA*	0.14 (0.10;0.18)	0.12 (0.08;0.20)	12.7 (2.26;71.0)	0.292	0.14 (0.11;0.20)	0.11 (0.07;0.18)	1.24 (0.11;13.7)	0.008
PV*	72.9 (49.6;105)	45.7 (33.5;64.8)	0.98 (0.97;0.98)	<0.001	69.3 (44.5;98.9)	41.7 (29.1;56.7)	0.97 (0.96;0.98)	<0.001
PSAD*	0.15 (0.10;0.23)	0.56 (0.25;1.46)	14.7 (7.56;28.8)	<0.001	0.16 (0.11;0.26)	0.65 (0.33;1.83)	109 (21.6;552)	<0.001
sagiPA*	20.1 (16.2;24.7)	15.5 (12.2;19.5)	0.89 (0.86;0.92)	<0.001	20.1 (16.3;24.8)	15.1 (12.1;18.0)	0.87 (0.82;0.92)	<0.001
sagiCGA*	12.8 (9.06;17.1)	8.09 (5.77;11.1)	0.83 (0.80;0.87)	<0.001	12.8 (9.62;17.1)	6.98 (5.16;9.99)	0.80 (0.74;0.86)	<0.001
sagiPZA*	6.49 (4.79;8.50)	7.11 (5.15;9.45)	1.06 (0.99;1.12)	0.041	6.23 (4.78;8.55)	6.80 (4.72;9.98)	1.08 (0.99;1.18)	0.187
transPA*	21.9 (17.5;28.0)	16.9 (13.0;20.4)	0.89 (0.86;0.91)	<0.001	21.0 (17.2;27.1)	16.1 (12.8;19.3)	0.86 (0.82;0.91)	<0.001
transCGA*	13.5 (9.76;18.2)	7.61 (5.77;10.6)	0.79 (0.75;0.83)	<0.001	13.9 (10.0;17.0)	7.06 (4.95;9.82)	0.78 (0.72;0.84)	<0.001
transPZA*	8.30 (5.91;10.3)	8.55 (5.96;10.9)	1.03 (0.98;1.07)	0.398	7.58 (5.89;9.77)	7.99 (6.30;10.5)	1.04 (0.96;1.13)	0.242
coroPA*	21.3 (17.1;27.9)	16.8 (13.1;20.2)	0.89 (0.86;0.91)	<0.001	22.2 (15.3;26.9)	15.5 (12.7;19.7)	0.88 (0.84;0.92)	<0.001
coroCGA*	16.2 (11.5;21.5)	8.91 (6.67;12.3)	0.83 (0.80;0.87)	<0.001	16.3 (10.7;21.3)	7.95 (5.58;11.4)	0.82 (0.77;0.87)	<0.001
coroPZA*	5.77 (4.23;7.21)	6.84 (5.31;8.79)	1.19 (1.11;1.28)	<0.001	5.45 (4.29;6.36)	6.76 (5.35;9.17)	1.33 (1.17;1.51)	<0.001
sagiPSAPA*	0.56 (0.39;0.88)	1.81 (0.73;4.44)	2.24 (1.84;2.73)	<0.001	0.56 (0.36;0.93)	2.02 (1.00;4.99)	3.13 (2.06;4.75)	<0.001
sagiPSACGA*	0.91 (0.62;1.37)	3.54 (1.46;8.17)	1.73 (1.51;1.97)	<0.001	0.82 (0.53;1.43)	4.10 (1.94;10.8)	2.30 (1.72;3.07)	<0.001
sagiPSAPZA*	1.78 (1.15;3.29)	4.18 (1.54;10.2)	1.19 (1.13;1.26)	<0.001	1.69 (0.99;2.95)	4.60 (2.30;10.0)	1.18 (1.09;1.27)	<0.001
transPSAPA*	0.51 (0.35;0.75)	1.55 (0.65;4.28)	2.48 (1.97;3.12)	<0.001	0.53 (0.34;0.81)	1.75 (0.84;4.81)	3.57 (2.22;5.76)	<0.001
transPSACGA*	0.81 (0.57;1.30)	3.43 (1.39;9.06)	1.75 (1.52;2.01)	<0.001	0.84 (0.54;1.40)	4.44 (1.78;11.9)	2.21 (1.67;2.93)	<0.001
transPSAPZA*	1.40 (0.89;2.36)	3.22 (1.29;8.44)	1.25 (1.17;1.34)	<0.001	1.44 (0.92;2.24)	3.85 (1.61;9.03)	1.32 (1.18;1.47)	<0.001
coroPSAPA*	0.52 (0.35;0.77)	1.47 (0.69;4.26)	2.51 (1.99;3.17)	<0.001	0.50 (0.34;0.82)	1.77 (0.88;4.44)	3.50 (2.21;5.53)	<0.001
coroPSACGA*	0.74 (0.47;1.09)	3.10 (1.29;7.36)	1.84 (1.58;2.14)	<0.001	0.66 (0.49;1.20)	4.08 (1.52;9.36)	2.39 (1.76;3.23)	<0.001
coroPSAPZA*	2.11 (1.21;3.57)	3.98 (1.72;9.83)	1.19 (1.13;1.25)	<0.001	2.08 (1.29;3.60)	4.80 (2.08;9.27)	1.23 (1.12;1.34)	<0.001
sagiPAI*	0.49 (0.34;0.74)	0.82 (0.51;1.26)	3.80 (2.51;5.75)	<0.001	0.47 (0.32;0.71)	0.85 (0.56;1.66)	4.22 (2.28;7.81)	<0.001
transPAI*	0.58 (0.41;0.87)	1.10 (0.62;1.61)	5.60 (3.71;8.46)	<0.001	0.54 (0.41;0.87)	1.27 (0.70;1.79)	7.70 (3.90;15.2)	<0.001
coroPAI*	0.36 (0.23;0.53)	0.74 (0.49;1.24)	9.21 (5.34;15.9)	<0.001	0.35 (0.23;0.48)	0.81 (0.55;1.32)	31.3 (10.7;92.0)	<0.001
ALP*	72.0 (60.0;85.0)	71.0 (59.0;88.3)	1.00 (1.00;1.01)	0.773	75.0 (60.5;85.0)	76.0 (61.0;89.0)	1.01 (1.00;1.01)	0.354
LDH*	176 (155;204)	182 (159;210)	1.01 (1.00;1.01)	0.034	179 (159;201)	184 (165;209)	1.01 (1.00;1.01)	0.125

PCa, prostate cancer; OR, odds ratio; FTPSA, free-to-total PSA; PA, prostate maximum sectional area; CGA, central gland sectional area; PZA, peripheral zone sectional area; PAI, PZA-to-CGA ratio; trans, transverse; coro, coronal; sagi, sagittal; PV, prostate volume; PSAD, PSA density.

*Continuous variables are shown as the median value and interquartile range.

All variables were not normally distributed.

**Table 2:** Clinical characteristics of patients in training cohort and validation cohort.

	All	Training cohort	Validation cohort	*p*-value
	(*n* = 691)	(*n* = 487)	(*n* = 204)	
Age*	69 (63;75)	69 (63;74)	70 (65;76)	0.047
BMI*	23.44 (21.50;25.51)	23.44 (21.50;25.56)	23.45 (21.51;25.50)	0.882
MRI:				0.827
Abnormal	397 (57.5%)	278 (57.1%)	119 (58.3%)	
Normal	294 (42.5%)	209 (42.9%)	85 (41.7%)	
FPSA*	1.53 (0.85;3.08)	1.59 (0.86;3.24)	1.46 (0.85;2.72)	0.244
PSA*	14.02 (8.70;35.09)	13.76 (8.56;34.65)	14.64 (9.63;35.76)	0.460
FTPSA*	0.13 (0.09;0.19)	0.13 (0.09;0.19)	0.13 (0.09;0.19)	0.742
PV*	57.1 (39.4;85.1)	58.3 (40.5;86.7)	55.0 (36.2;82.5)	0.192
PSAD*	0.24 (0.13;0.65)	0.23 (0.13;0.65)	0.26 (0.14;0.65)	0.285
sagiPA*	17.9 (13.9;22.3)	18.0 (13.9;22.5)	17.5 (13.6;22.2)	0.533
sagiCGA*	10.4 (6.86;14.8)	10.4 (6.92;15.0)	10.4 (6.69;14.4)	0.548
sagiPZA*	6.71 (4.84;9.04)	6.74 (4.88;9.05)	6.59 (4.72;8.86)	0.532
transPA*	19.0 (15.0;24.2)	19.3 (15.3;24.3)	18.6 (14.9;23.7)	0.304
transCGA*	10.3 (7.05;15.2)	10.5 (7.21;15.2)	10.2 (6.62;15.1)	0.424
transPZA*	8.20 (5.95;10.7)	8.37 (5.94;10.8)	7.82 (6.01;10.3)	0.386
coroPA*	18.8 (14.5;24.6)	18.9 (14.7;24.6)	18.0 (14.2;24.4)	0.298
coroCGA*	12.0 (7.91;17.8)	12.1 (8.11;17.7)	11.4 (7.68;17.9)	0.481
coroPZA*	6.13 (4.58;7.92)	6.30 (4.54;7.97)	5.88 (4.64;7.65)	0.187
sagiPSAPA*	0.83 (0.48;2.07)	0.81 (0.47;1.99)	0.93 (0.49;2.19)	0.525
sagiPSAPZA*	2.34 (1.28;6.03)	2.29 (1.26;6.02)	2.47 (1.32;6.04)	0.635
sagiPSACGA*	1.43 (0.76;3.96)	1.37 (0.77;3.70)	1.53 (0.72;4.04)	0.560
transPSAPA*	0.70 (0.43;1.80)	0.69 (0.43;1.79)	0.75 (0.44;1.83)	0.367
transPSAPZA*	1.90 (1.07;4.27)	1.86 (1.02;4.20)	1.99 (1.19;4.40)	0.365
transPSACGA*	1.35 (0.73;4.04)	1.28 (0.73;3.77)	1.41 (0.75;4.10)	0.479
coroPSAPA*	0.73 (0.43;1.82)	0.72 (0.43;1.74)	0.78 (0.44;2.00)	0.386
coroPSAPZA*	2.57 (1.44;5.79)	2.57 (1.35;5.65)	2.54 (1.62;6.06)	0.371
coroPSACGA*	1.13 (0.62;3.52)	1.10 (0.62;3.38)	1.20 (0.62;3.69)	0.563
coroPAI*	0.50 (0.31;0.86)	0.50 (0.32;0.85)	0.48 (0.29;0.88)	0.779
transPAI*	0.75 (0.48;1.28)	0.75 (0.49;1.28)	0.76 (0.45;1.30)	0.983
sagiPAI*	0.60 (0.41;1.00)	0.60 (0.42;0.99)	0.57 (0.38;1.07)	0.812
ALP*	73.0 (60.0;86.3)	71.7 (59.0;86.0)	76.0 (60.8;87.0)	0.287
LDH*	179 (157;206)	178 (156;207)	181 (162;205)	0.490

PCa, prostate cancer; OR, odds ratio; FTPSA, free-to-total PSA; PA, prostate maximum sectional area; CGA, central gland sectional area; PZA, peripheral zone sectional area; PAI, PZA-to-CGA ratio; trans, transverse; coro, coronal; sagi, sagittal; PV, prostate volume; PSAD, PSA density.

*Continuous variables are shown as the median value and interquartile range.

All variables were not normally distributed.

### Multivariate Logistic Regression Models

To evaluate the synergistic ability of every single predictor for predicting PCa, we created different models that did not contain multicollinearity. The first three models with the highest AUC were chosen as our final models, which were model 1, model 2, and model 3. Model 1 consists of age, PSA, MRI, transCGA, and coroPZA after excluding sagiPAI, PV, and PSAD. Model 2 consists of age, PSA, MRI, transPAI, coroPZA, and transPA after excluding PV and PSAD. Model 3 consists of age, PSA, MRI, transPAI, and PV after excluding sagiPAI, coroPAI, and PSAD (**Table 3**). Age, MRI, and PSA were independent predictors of PCa in three models. The statistical significance of PV was not detected in both model 1 and model 2. The statistical significance of PSAD was not detected in all models.

**Table 3:** Multivariate logistic regression analysis of predictors associated with PCa before the prostate biopsy.

	Model 1				Model 2				Model 3		
Parameters	Coefficient	OR (95% CI)	*p*-value	Parameters	Coefficient	OR (95% CI)	*p*-value	Parameters	Coefficient	OR (95% CI)	*p*-value
Age	0.070	1.073 (1.037–1.112)	0.0001	Age	0.071	1.073 (1.038–1.113)	0.0001	Age	0.071	1.074 (1.039–1.112)	<0.0001
MRI	−1.396	0.248 (0.144–0.419)	<0.0001	MRI	−1.400	0.247 (0.144–0.417)	<0.0001	MRI	−1.304	0.271 (0.160–0.455)	<0.0001
PSA	0.045	1.046 (1.021–1.072)	0.0002	PSA	0.044	1.045 (1.021–1.069)	0.0002	PSA	0.045	1.046 (1.023–1.070)	0.0001
sagiPAI	0.419	1.521 (0.884–2.682)	0.1403	transPAI	1.511	4.533 (2.587–8.246)	<0.0001	sagiPAI	0.339	1.404 (0.794–2.482)	0.2404
coroPZA	0.149	1.161 (1.040–1.298)	0.0063	coroPZA	0.150	1.162 (1.043–1.312)	0.0093	transPAI	1.198	3.313 (1.878–6.034)	0.0001
transCGA	−0.306	0.736 (0.655–0.822)	<0.0001	transPA	−0.168	0.845 (0.771–0.923)	0.0003	coroPAI	0.321	1.378 (0.918–2.377)	0.1223
PV	0.007	1.007 (0.990–1.023)	0.4148	PV	0.002	1.002 (0.984–1.020)	0.7963	PV	−0.022	0.978 (0.967–0.988)	<0.0001
PSAD	−0.321	0.726 (0.326–2.169)	0.4816	PSAD	−0.323	0.724 (0.329–2.082)	0.4663	PSAD	−0.29	0.748 (0.345–2.114)	0.5057

PCa, prostate cancer; OR, odds ratio; PA, prostate maximum sectional area; CGA, central gland sectional area; PZA, peripheral zone sectional area; PAI, PZA-to-CGA ratio; trans, transverse; coro, coronal; sagi, sagittal; PV, prostate volume; PSAD, PSA density.

### Comparison of Predictive Accuracy

**Figure 3:**
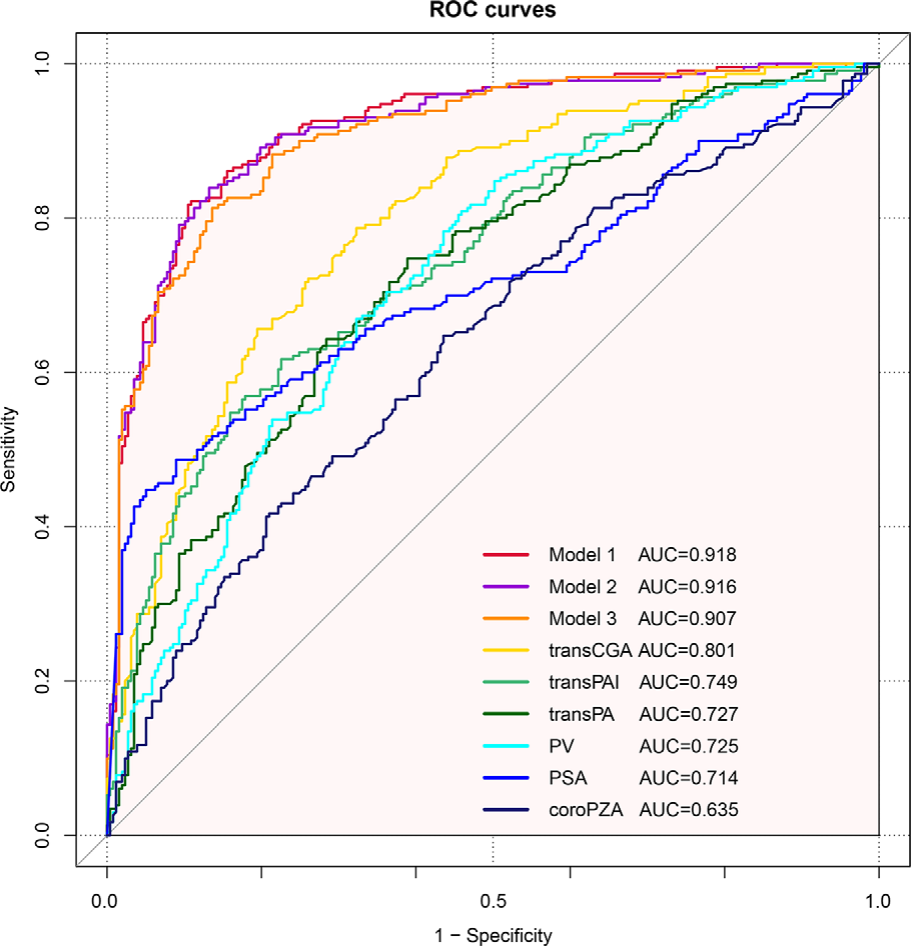
Receiver operating characteristic curves depicting the accuracy of predictors of PCa before the initial biopsy. Base model: age + PSA + MRI. Model 1: Base model + coroPZA + transCGA. Model 2: Base model + transPAI + coroPZA + transPA. Model 3: Base model + transPAI + PV.

Furthermore, predictive accuracy of each predictor alone and models were assessed separately with ROC curve analysis. The AUC of model 1 (base model + coroPZA + transCGA) for predicting PCa was the highest among the models of any single predictor alone and the base model combined with any other predictors (**Figure 3** and **Table 4**). Delong test was used to compare the statistical difference of the AUC among three models and single predictors. Compared with model 3 (AUC = 0.907), the AUC of model 1 (AUC = 0.918) and model 2 (AUC = 0.916) had the same higher statistical advantages in the training cohort, while no statistical difference was found among the three models in the validation cohort (**Table 5**). The AUC of transCGA (0.801) was significantly higher than other single predictors. The AUC of coroPZA (0.635) was lower than other predictors, while there was no significant difference of AUC among transPAI, transPA, PV, and PSA in the training cohort (**Table 6**).

**Table 4:** The AUC and cutoff values for predicting biopsy outcome and their sensitivity, specificity, and positive and negative likelihood ratios for PCa.

Parameters	AUC	Cutoff value	Sensitivity (%)	Specificity (%)	Positive likelihood ratio	Negative likelihood ratio
coroPZA	0.635	6.055	64.8%	56.4%	1.49	0.62
PSA	0.714	28.775	48.7%	91.1%	5.47	0.56
PV	0.725	69.562	80.9%	54.1%	1.76	0.35
transPA	0.727	18.505	64.3%	71.6%	2.26	0.50
transPAI	0.749	0.906	61.7%	77.4%	2.73	0.49
transCGA	0.801	11.045	78.7%	67.7%	2.44	0.31
Model 1	0.918	0.525	82.2%	89.1%	6.36	0.19
Model 2	0.916	0.471	83.9%	86.8%	7.75	0.27
Model 3	0.907	0.480	81.3%	86.4%	5.98	0.22

PCa, prostate cancer; AUC, area under the curve; PA, prostate maximum sectional area; CGA, central gland sectional area; PZA, peripheral zone sectional area; PAI, PZA-to-CGA ratio; trans, transverse; coro, coronal; PV, prostate volume; Base model, Age + PSA + MRI; Model 1, Base model + coroPZA + transCGA; Model 2, Base model + transPAI + coroPZA + transPA; Model 3, Base model + transPAI + PV.

**Table 5:** The statistical difference in AUC of predicting PCa among three models.

Comparison (*p*-value)by Delong test	Model 1 *vs.* Model 2	Model 1 *vs.* Model 3	Model 2 *vs.* Model 3
Training cohort	0.300	0.019	0.042
Validation cohort	0.706	0.293	0.150

PCa, prostate cancer; Base model, Age + PSA + MRI; Model 1, Base model + coroPZA + transCGA; Model 2, Base model + transPAI + coroPZA + transPA; Model 3, Base model + transPAI + PV.

**Table 6:** The statistical difference in AUC of predicting PCa among single predictors in the training cohort.

Comparison (*p*-value) by Delong test	transCGA	transPAI	transPA	PV	PSA	coroPZA
transCGA	—					
transPAI	0.002	—				
transPA	<0.001	0.442	—			
PV	<0.001	0.359	0.843	—		
PSA	0.01	0.307	0.718	0.762	—	
coroPZA	<0.001	<0.001	0.017	0.02	0.018	—

PCa, prostate cancer; PA, prostate maximum sectional area; CGA, central gland sectional area; PZA, peripheral zone sectional area; PAI, PZA-to-CGA ratio; trans, transverse; coro, coronal; PV, prostate volume.

### Nomograms, Calibration Plots, and DCA Curves

**Figure 4:**
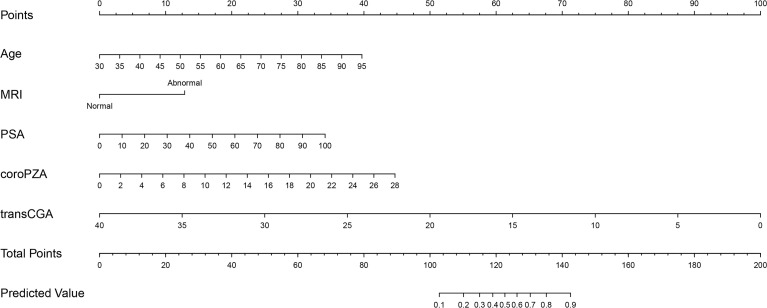
Nomogram predicting the probability of PCa at the initial biopsy based on model 1.

**Figure 5:**
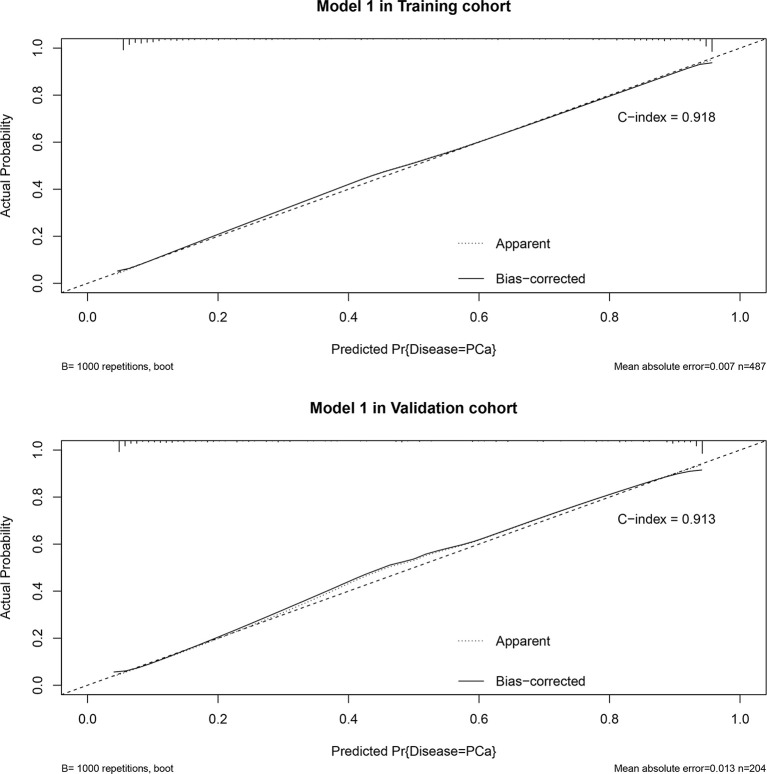
Calibration plot in training cohort and validation cohort and predictive accuracy for PCa at initial biopsy based on model 1.

**Figure 6:**
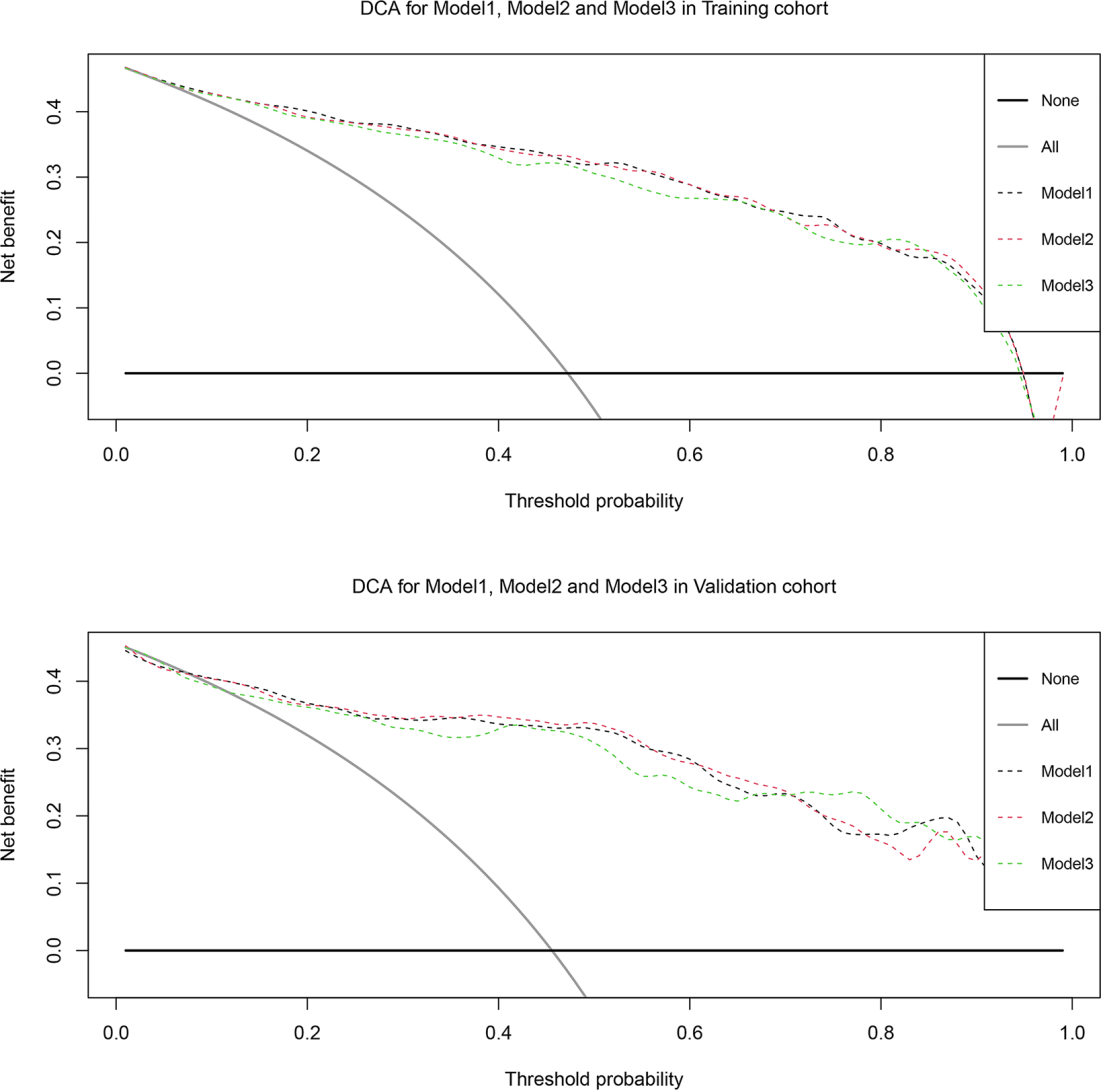
Decision curve analysis of the effect of the nomogram based on model 1 for predicting prostate cancer in training cohort and validation cohort. Net benefit of nomogram is plotted with threshold probabilities for prostate cancer compared with the strategies of treating all patients or no one. The decision curve illustrated net benefit was improved when threshold probability > 8%.

Based on the multivariate regression coefficients, the predictive models were visually presented as nomograms (**Figure 4** and **Figure S3**). The nomogram’s discrimination of three models in the training cohort and validation cohort was shown in the calibration plot (**Figure 5**, **Figures S4** and **S5**). The C-index of model 1 for predicting PCa was 0.918 in the training cohort. The performance of model 2 (0.916) and model 3(0.907) in the calibration plot was not as good as that of model 1 in the training cohort, which demonstrates the superior fit of model 1. DCA curves showed that the nomogram based on model 1 has better net benefit gains in all range of threshold probabilities in the training cohort, while net benefit gains only improved when threshold probabilities are >8% in the validation cohort (**Figure 6**).

## Discussion

PCa is a malignant form of cancer whose diagnosis depends on the histopathological verification of adenocarcinoma in a prostate biopsy. However, excess of prostate biopsy has led to increased side effects such as bleeding and infection. It also caused the inferior positive rate of 30%–40% (3, 5). A retrospective study including 1,203 patients who underwent prostate biopsy demonstrated that the overall rates of infectious and hemorrhagic complications after prostate biopsy were 8.23% and 15.71%, respectively (16). So, it needs to establish a method to carefully select patients who need prostate biopsy.

Numerous studies have reported the predictive value of prostate volume (PV) and prostate volume-adjusted PSA (i.e., PSAD) for PCa. For example, one study that measured 235 patients’ prostate volume and PSA levels demonstrated that the AUC values of PSAD (0.712) and prostate volume (0.710) were higher than that of PSA (0.517) for diagnosing PCa (17). Our previous research found that the utility of PSAD for performing surveillance in patients at risk of PCa was higher than that of standard variables such as PSA (18). However, a retrospective study found that PSAD and PSA (AUC = 0.620 and 0.530, respectively) failed to outperform prostate volume (AUC = 0.680) for preoperative prediction of PCa (19). The current study confirmed that both PV and PSAD were good predictors of PCa in univariate logistic analysis. However, none of them showed statistical significance in model 1 and model 2. The reason is possibly that the prostate is not a regular geometric solid especially in the malignant growth mode of the tumor. Furthermore, prostate volume is usually estimated by an elliptical sphere formula (PV = 0.52 × length × width × height). Any error on the length, width, or height of prostate may be magnified through the multiplication (20). Previous studies had shown a high magnitude of bias between the calculation of prostate volume by the prolate ellipsoid formula and the actual prostate volume, which casts doubt on the diagnostic efficacy of PV and PSAD in PCa (21). Some studies had confirmed that the bias of calculated prostate volume fluctuates between 10% and 20% (8, 9). As we mentioned above, the increase of PSA level caused by any other reason, except for PCa, may lead to the error of PSAD and reduce its specificity for predicting PCa. Therefore, we do not think that PV and PSAD have the leading advantage of predicting PCa.

In order to overcome the difficulties mentioned above, we sought to replace the role of prostate volume with the incorporation of more accurate, simple prostatic imaging parameters. We found that PA (prostate maximum sectional area) is a good prostatic imaging parameter for predicting PCa by MRI test in line with the above requirements. MRI has higher spatial resolution and better soft tissue contrast than TRUS, and MRI can provide more accurate PA. So, it can reflect the actual size of the prostate (19). Thus, we used MRI-based PA as an alternative predictor of prostate volume for predicting PCa. As far as we know, we are the first to use prostate maximum sectional areas in sagittal, transverse, and coronal directions to predict PCa. The data of prostate sectional area from three different directions may help to improve its representativeness for irregular prostate. It can possibly find out the shape characteristics of the prostate in different directions. On the other hand, the area and its zone area of prostate were actually measured in MRI segments, which will decrease the systematic error to a great extent compared with the calculated prostate volume by formula. In our research, all sectional area predictors have statistical differences between PCa and no PCa patients in univariate analysis, except for transPZA. It proved that they had great potential in predicting PCa. We found that BPH patients had larger PA and CGA but smaller PZA in three directions compared with those who had PCa. It might result from the fact that BPH contributes to mechanical stress fields by pathological enlargement of the prostate central gland, hence further restraining PCa growth, as PCa mostly originates in the peripheral zone of the prostate (22). Through Delong test among single predictors, we find out the transCGA has the significantly highest AUC (0.801) among all predictors. Compared with no PCa, transCGA is significantly smaller in patients with PCa (*p* < 0.001). We speculated that this is due to the special behavior pattern of PCa growth on the transverse section. So far, we have not seen any relevant report that needs to be confirmed by further pathological or anatomical studies.

To further explore the potential of prostate sectional area related predictors to predict PCa, we built new predictors like PAI, PSAPA, PSACGA, and PSAPZA (calculated by PZA/CGA, PSA/PA, PSA/CGA, and PSA/PZA) based on the sectional area from three different directions in MRI segments. Each of these predictors had the potential ability to distinguish PCa from no PCa in univariate logistic analysis. Unfortunately, we found that PSAPA, PSACGA, and PSAPZA failed to outperform any predictors when we discovered the different models. So, we did not include these predictors in multivariate logistic analysis. However, PAI (prostate area index) showed favorable predictability in our final model, especially transPAI, which had an AUC (0.749) second only to transCGA. In both model 2 and model 3, transPAI had a certain contribution to the diagnosis of PCa compared with coroPAI and sagiPAI. This also confirms our previous hypothesis about the special behavior pattern of PCa growth on the transverse section. Patients with PCa have higher transPAI. It may be due to PCa often originating in the peripheral zone, which causes the enlargement of PZA. Then, it leads to an increase in PAI (PZA-to-CGA ratio) in turn.

With the changes to people’s living habits, and the progression of population aging, the prevalence of PCa is increasing annually, especially among adults aged over 70 years. This situation has seriously affected the health of older adults (23). Our results also confirm this conclusion. The median age of men with PCa was 75 years, compared with 69 years for men without PCa (*p* < 0.001). Thus, age can be used as a reference for prostate biopsy.

To reduce unnecessary prostate biopsy and improve the diagnostic accuracy of PCa in clinical practice, nomograms integrating many independent predictors of PCa have been developed and validated. A previous study reported that nomograms could provide more individualized risk estimations of a certain disease, which could help clinicians to make management-related decisions for patients with PCa (24). For example, a nomogram developed on the basis of 1,144 men who underwent TRUS found that the C-index (0.876) was associated with their best model that integrates age, PSA, percentage free PSA, DRE, prostate transition zone volume, and TRUS for predicting PCa (25). Another study integrated age, prostate volume, PSA, FTPSA, TRUS, and DRE as its best model to develop a nomogram for the probability of detecting PCa in all patients, achieving a C-index of 0.853 (26). In the current study, we chose the best prediction model of the base model + transCGA + coroPZA to construct a new nomogram that could provide the risk of PCa for individual Chinese patients. Internal validation showed a predictive accuracy (C-index = 0.913) for PCa, which gained an advantage over some previous nomograms developed by Chinese researchers.

Our study has certain limitations. As with any retrospective study, there was the risk of selection bias in assessing the value of the prostate maximum sectional area on mp-MRI. In addition to the issues surrounding the small sample size, our models were calibrated using internal validation only, with no external validation conducted to ensure their utility. Therefore, further clinical studies that employ long-term follow-up to evaluate our model’s practical applicability are required before it is prospectively applied to patients.

We first found that MRI-based CGA, PZA, and PA in the sagittal, transverse, and coronal section have potential predictive value for diagnosing PCa, especially transCGA. We put forward a new predictor named transPAI. We found that it was significantly higher in PCa patients. Our nomogram model based on age + MRI + PSA + transCGA + coroPZA had great predictive accuracy for PCa. The application of this nomogram model may further decrease the rate of unnecessary biopsies.

## Data Availability Statement

Publicly available datasets were analyzed in this study. These data can be found here: https://www.wolai.com/nYw7M5iJDyxn91RDWTmoPj?theme=light.

## Author Contributions

SJ conceived the study and carried out the investigation, methodology, and original draft preparation. ZH and BL carried out the investigation, methodology, and formal analysis, and participated in the conceptualization of the manuscript. ZC carried out the data curation, investigation, and methodology. YX, WZ, and YW participated in the formal analysis. ML conceived the study; carried out the supervision, review, and editing of the manuscript; and took responsibility for the integrity of the work as corresponding author. All authors contributed to the article and approved the submitted version.

## Funding

This work was supported by the Startup Fund for Scientific Research, Fujian Medical University (Grant number: 2018QH1044) and Joint Funds for the Innovation of Science and Technology, Fujian province (Grant number: 2017Y9023).

## Conflict of Interest

The authors declare that the research was conducted in the absence of any commercial or financial relationships that could be construed as a potential conflict of interest.

## Publisher’s Note

All claims expressed in this article are solely those of the authors and do not necessarily represent those of their affiliated organizations, or those of the publisher, the editors and the reviewers. Any product that may be evaluated in this article, or claim that may be made by its manufacturer, is not guaranteed or endorsed by the publisher.

## Supplementary Material

Supplementary Figure 1 Prostate location and MR imagine in transverse **(A)**, sagittal **(B)** and coronal **(C)** plane. Red: Central gland sectional area, CGA; Blue: Peripheral zone sectional area, PZA.

Supplementary Figure 2 Correlation analysis among each predictor used to eliminate combinations including collinear predictors.

Supplementary Figure 3 Nomogram predicting the probability of PCa at the initial biopsy based on model 2 **(A)** and model 3 **(B)**.

Supplementary Figure 4 Calibration plot in training cohort and validation cohort and predictive accuracy for PCa at initial biopsy based on model 2.

Supplementary Figure 5 Calibration plot in training cohort and validation cohort and predictive accuracy for PCa at initial biopsy based on model 3.

## Abbreviations

BMI, body mass index; FPSA, free PSA; PSA, prostate-specific antigen; FTPSA, $\frac{\text{free PSA}}{\text{PSA}};$ PV, prostate volume; sagiPSAPA, $\frac{\text{PSA}}{\text{sagi PA}};$ PSAD, PSA density; sagiPSACGA, $\frac{\text{PSA}}{\text{sagi CGA}};$ PA, prostate maximum sectional area; sagiPSAPZA, $\frac{\text{PSA}}{\text{sagi}\;\text{PZA}};$ CGA, central gland sectional area; transPSAPA, $\frac{\text{PSA}}{\text{trans PA}};$ PZA, peripheral zone sectional area; transPSACGA, $\frac{\text{PSA}}{\text{trans CGA}};$ sagiPA, sagittal prostate maximum sectional area; transPSAPZA, $\frac{\text{PSA}}{\text{trans PZA}};$ sagiCGA, sagittal central gland sectional area; coroPSAPA, $\frac{\text{PSA}}{\text{coro PA}};$ sagiPZA, sagittal peripheral zone sectional area; coroPSACGA, $\frac{\text{PSA}}{\text{coro CGA}};$ transPA, transverse prostate maximum sectional area; coroPSAPZA, $\frac{\text{PSA}}{\text{coro PZA}};$ transCGA, transverse central gland sectional area; sagiPAI, $\frac{\text{sagiPZA}}{\text{sagi}\;\text{CGA}};$ transPZA, transverse peripheral zone sectional area; transPAI, $\frac{\text{transPZA}}{\text{trans CGA}};$ coroPA, coronal prostate maximum sectional area; coroPAI, $\frac{\text{coroPZA}}{\text{coro CGA}};$ coroCGA, coronal central gland sectional area; ALP, alkaline phosphatase; coroPZA, coronal peripheral zone sectional area; LDH, lactate dehydrogenase
