# Supplementary figures and images for: MRI-Based Nomogram of Prostate Maximum Sectional Area and Its Zone Area for Prediction of Prostate Cancer
Source: Front Oncol. 2021 Sep 9;11:708730. doi: 10.3389/fonc.2021.708730 (PMC8458948; doi:10.3389/fonc.2021.708730)

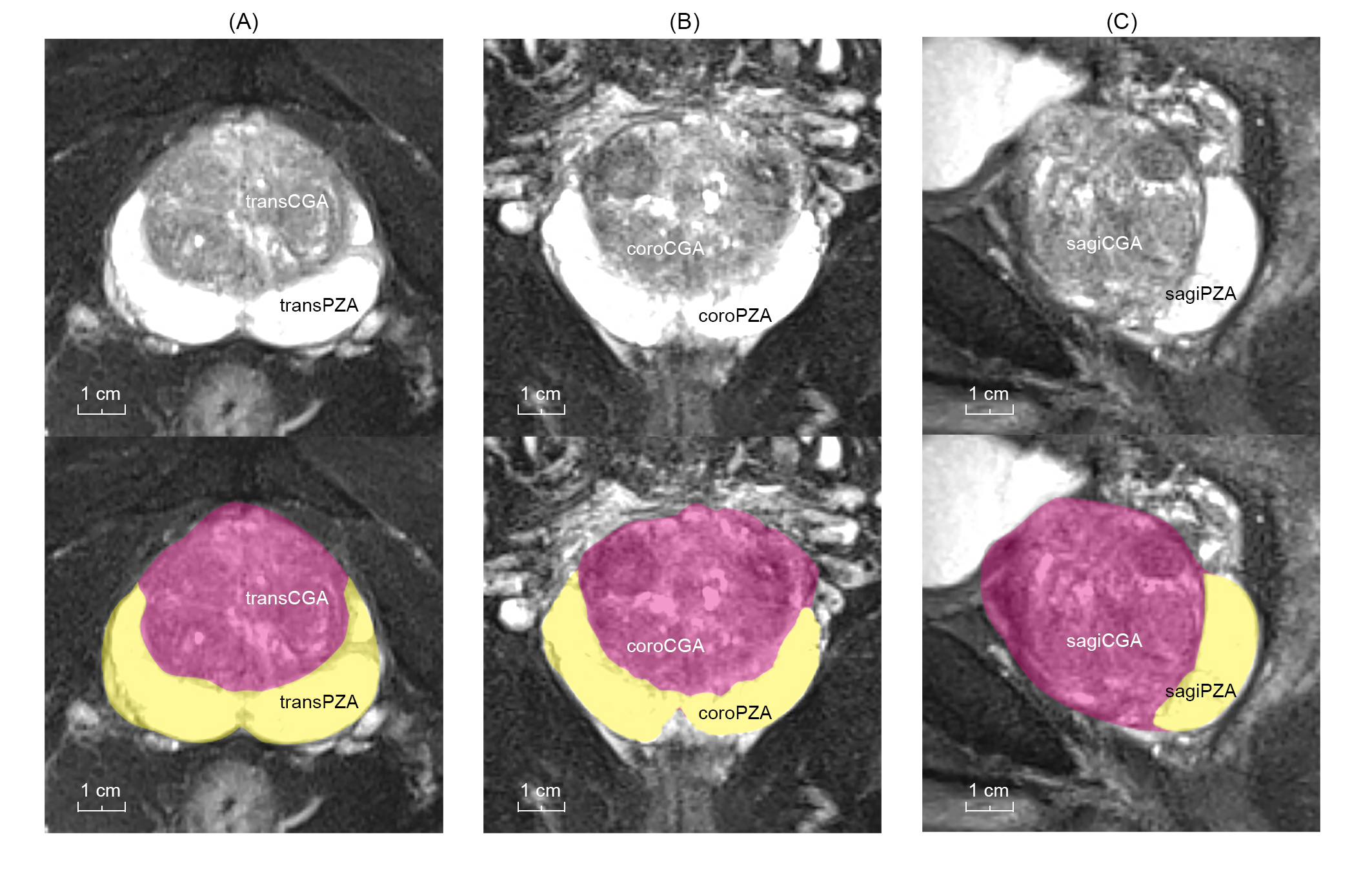

Supplement: Supplementary Figure 1 — Prostate location and MR imagine in transverse (A), sagittal (B) and coronal (C) plane. Red: Central gland sectional area, CGA; Blue: Peripheral zone sectional area, PZA. [file Presentation_1.zip › Supplementary materials/Figure S1.tif]

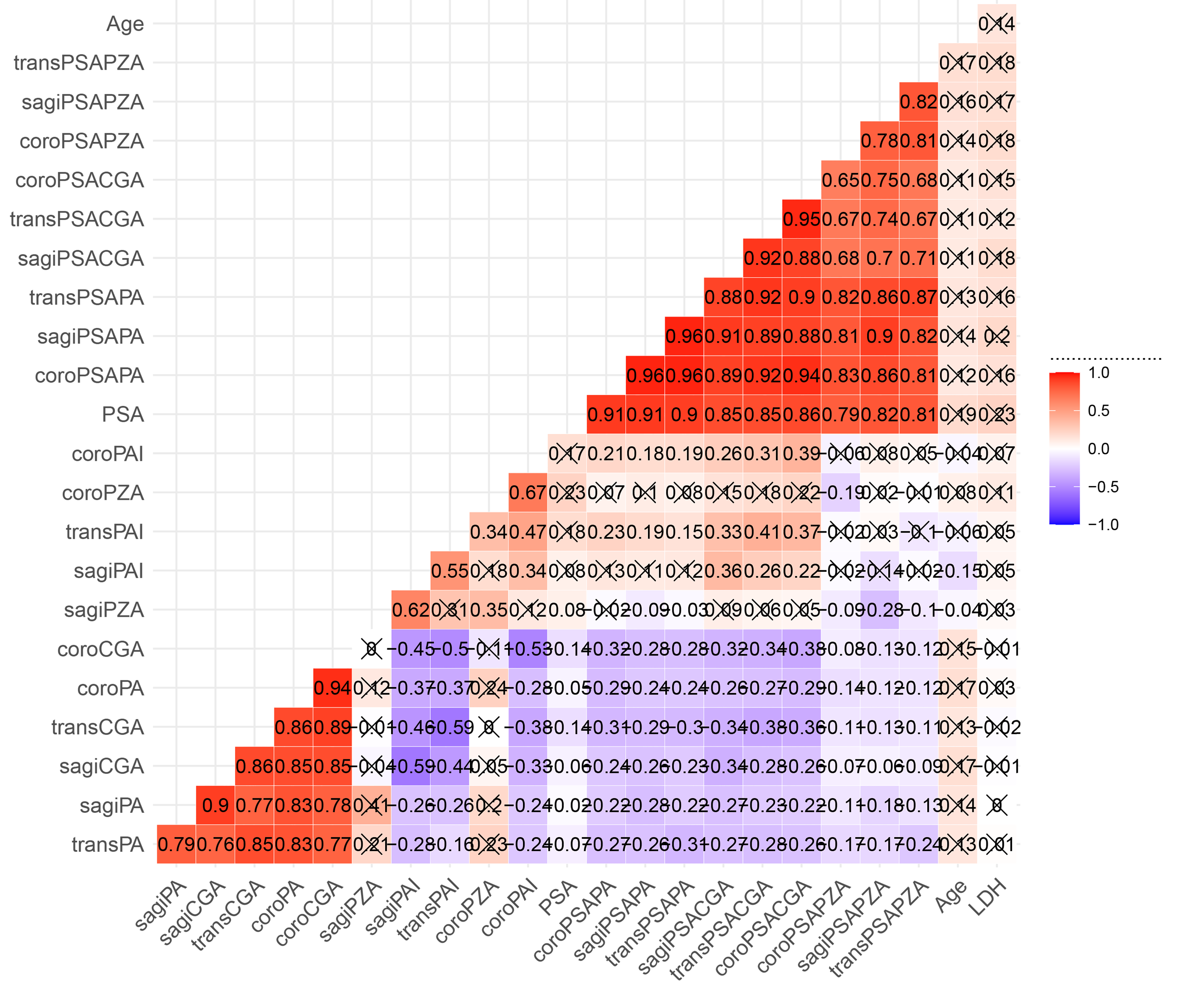

Supplement: Supplementary Figure 1 — Prostate location and MR imagine in transverse (A), sagittal (B) and coronal (C) plane. Red: Central gland sectional area, CGA; Blue: Peripheral zone sectional area, PZA. [file Presentation_1.zip › Supplementary materials/Figure S2.tif]

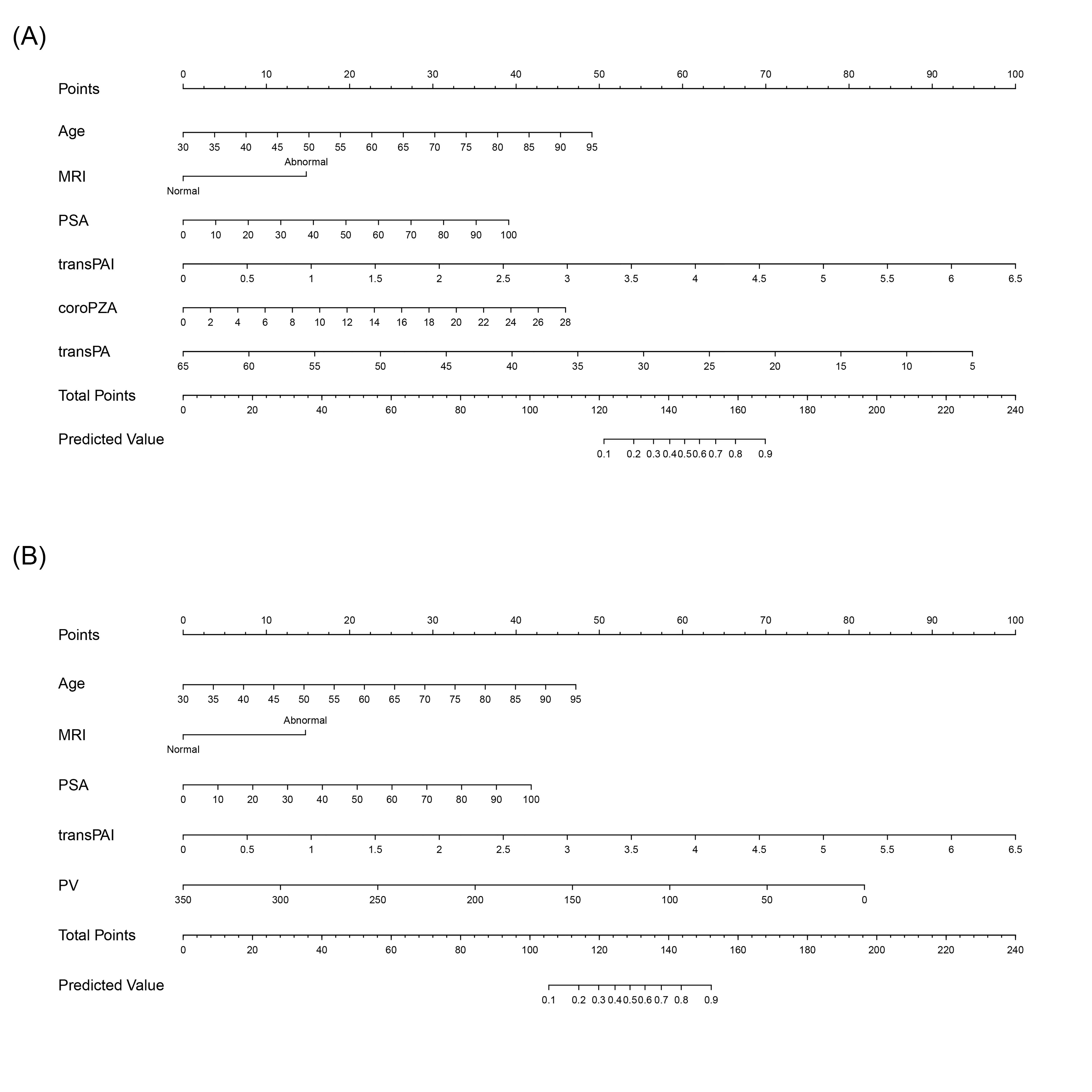

Supplement: Supplementary Figure 1 — Prostate location and MR imagine in transverse (A), sagittal (B) and coronal (C) plane. Red: Central gland sectional area, CGA; Blue: Peripheral zone sectional area, PZA. [file Presentation_1.zip › Supplementary materials/Figure S3.tif]

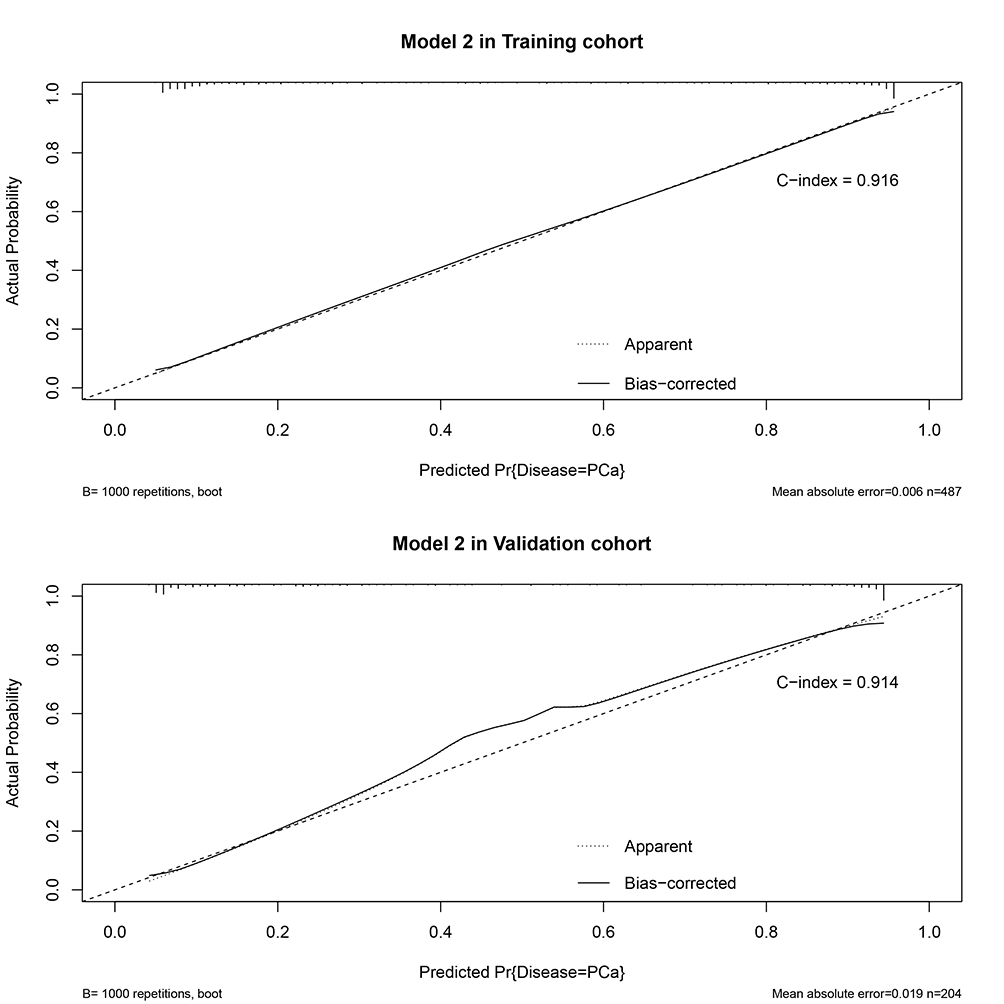

Supplement: Supplementary Figure 1 — Prostate location and MR imagine in transverse (A), sagittal (B) and coronal (C) plane. Red: Central gland sectional area, CGA; Blue: Peripheral zone sectional area, PZA. [file Presentation_1.zip › Supplementary materials/Figure S4.tif]

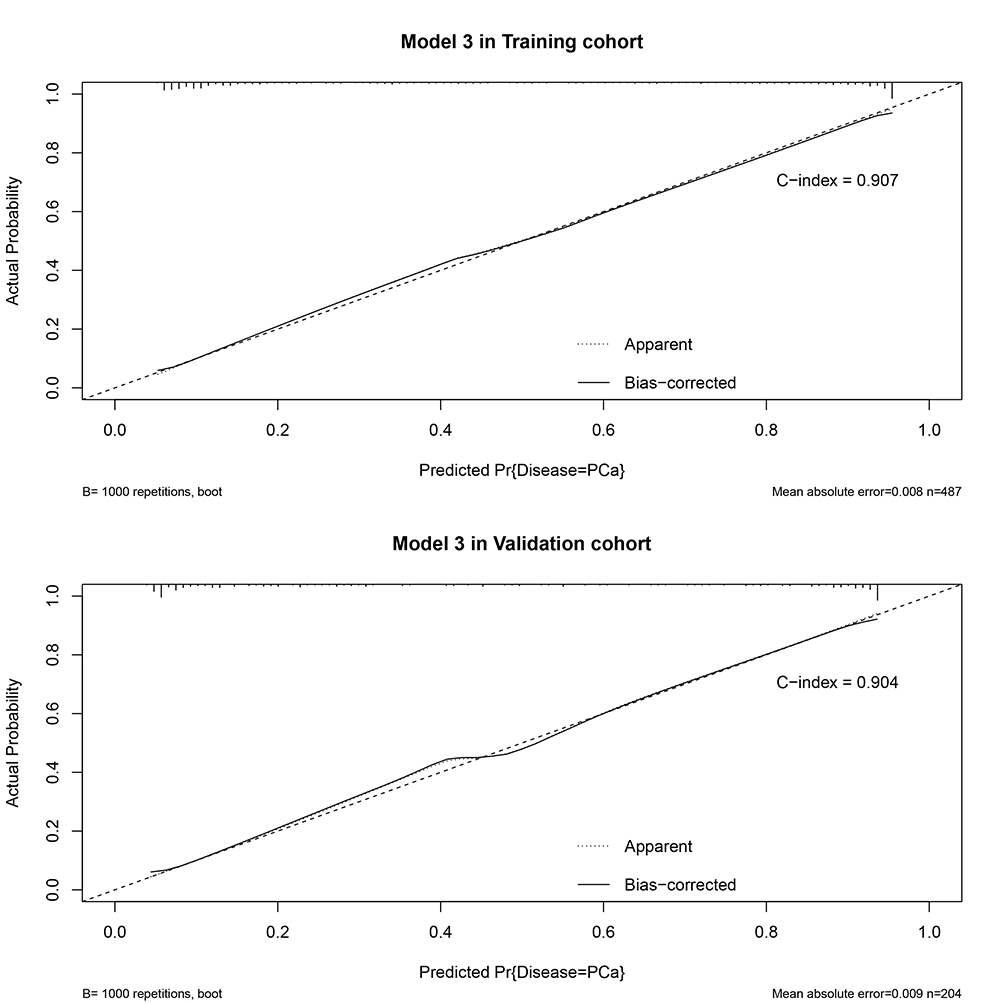

Supplement: Supplementary Figure 1 — Prostate location and MR imagine in transverse (A), sagittal (B) and coronal (C) plane. Red: Central gland sectional area, CGA; Blue: Peripheral zone sectional area, PZA. [file Presentation_1.zip › Supplementary materials/Figure S5.tif]
